# Combination of Cystatins 9 and C Modulates Serum Biomarkers Associated with Inflammation and Provides Prophylactic as Well as Long-Term Protection against Multidrug-Resistant *Klebsiella pneumoniae*

**DOI:** 10.1128/AAC.02519-18

**Published:** 2019-04-25

**Authors:** Alex J. Holloway, JiehJuen Yu, Bernard P. Arulanandam, Gregg N. Milligan, Tonyia D. Eaves-Pyles

**Affiliations:** a Department of Microbiology and Immunology, University of Texas Medical Branch, Galveston, Texas, USA; b The South Texas Center for Emerging Infectious Diseases, Department of Biology, University of Texas at San Antonio, San Antonio, Texas, USA; c Department of Pediatrics, University of Texas Medical Branch, Galveston, Texas, USA

**Keywords:** NDM-1 *Klebsiella pneumoniae*, adoptive B-cell transfer, amyloid A, cystatin 9, cystatin C, immunomodulation, immunotherapy, inflammation, multidrug resistant, pneumonia

## Abstract

We have identified recombinant human cystatins 9 (rCST9) and C (rCSTC) as a combination immunotherapeutic treatment against multidrug-resistant (MDR) New Delhi metallo-β-lactamase-1 (NDM-1)-producing Klebsiella pneumoniae. We evaluated the lasting protection of rCST9/rCSTC treatment against MDR NDM-1 K. pneumoniae pneumonia. Results showed that rCST9/rCSTC treatment modulated endogenous serum biomarkers, cystatins 9 and C and amyloid A, associated with poor patient outcomes and provided prophylactic and long-term protection in a murine model of pneumonia.

## Abstract (precis)

We have identified recombinant human cystatins 9 (rCST9) and C (rCSTC) as a combination immunotherapeutic treatment against multidrug-resistant (MDR) New Delhi metallo-β-lactamase-1 (NDM-1)-producing Klebsiella pneumoniae. We evaluated the lasting protection of rCST9/rCSTC treatment against MDR NDM-1 K. pneumoniae pneumonia.

## INTRODUCTION

The frequency of multidrug (MDR) resistance among pathogenic bacteria, such as New Delhi metallo-β-lactamase-1 (NDM-1) Klebsiella pneumoniae-induced pneumonia, and the ability of the bacteria to continually evolve in a manner that renders traditional antibiotic treatment ineffective are growing health care concerns around the world. Development and implementation of successful treatment alternatives to combat MDR pathogens are currently lacking. To address this gap, we discovered that coadministration of human recombinant human cystatins 9 (rCST9) and C (rCSTC) is an effective immunomodulatory therapy that provides unprecedented survival outcomes against pneumonia induced by an intranasal (i.n.) challenge with MDR NDM-1 K. pneumoniae (1). Cystatins are cysteine protease inhibitors found throughout the body tissues and fluids (2, 3). These naturally produced proteins maintain a balance among cysteine proteinases to prevent the excessive breakdown of the extracellular matrix, leading to dysregulated immune responses and tissue damage (2–5). During pathophysiological events, an imbalance between endogenous cystatin levels and cysteine proteinases can develop, resulting in dysregulated inflammation (5–7).

Our findings have shown that rCST9 treatment modulated damaging inflammation leading to significant improvement in survival in a murine model of tularemia (8). Similarly, we recently reported that i.n. coadministration of rCST9/rCSTC (50 pg of each/mouse) 1 h postinfection (p.i.) followed by a subsequent rCST9/rCSTC dose (500 pg of each/mouse) given intraperitoneally (i.p.) at 3 days p.i. significantly modulated excessive inflammation, decreased apoptosis, preserved the structural integrity of the lung, decreased bacterial load, and significantly increased survival outcomes in our murine model of pneumonia induced by MDR NDM-1 K. pneumoniae (1). A single one-time dose of rCST9/rCSTC (500 pg of each) given i.p. at 3 days p.i. also afforded significant protection against NDM-1 K. pneumoniae-induced pneumonia that was nearly equivalent to the two doses of rCST9/rCSTC (1).

**FIG 1:**
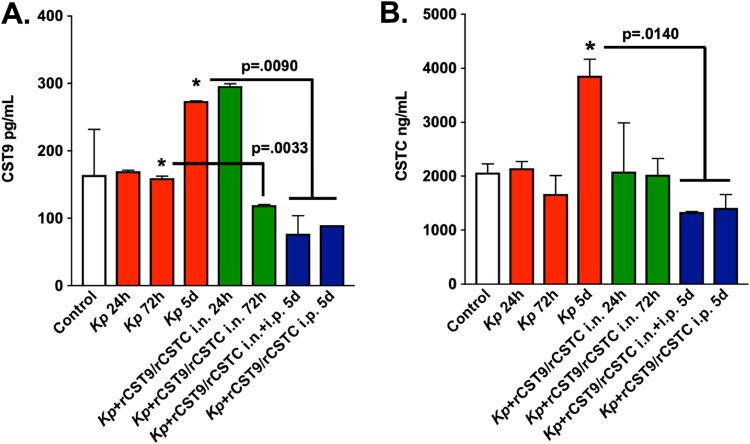
rCST treatment modulated endogenous serum CST9 and CSTC levels. (A) Both optimal rCST9/rCSTC treatment regimens significantly modulated endogenous CST9 serum levels by 72 h (*P* = 0.0033) and 5 days (*P* = 0.0090) in treated mice compared with untreated MDR NDM-1 K. pneumoniae-infected mice. (B) Likewise, endogenous serum CSTC levels were significantly decreased by 5 days p.i. in treated mice compared with untreated infected mice on day 5 p.i. (*P* = 0.0140). White bars, untreated/uninfected controls; red bars, untreated infected mice; green bars, infected mice receiving rCST9/rCSTC i.n. treatment (50/50 pg) 1 h p.i.; blue bars, infected mice receiving rCST9/rCSTC i.n. treatment (50/50 pg) 1 h p.i. and/or rCST9/rCSTC i.p. (500/500 pg) at 3 days p.i. Data are presented as means ± SEM. *, *P* < 0.05.

Because high endogenous serum CSTC levels serve as biomarkers that are strongly linked to poor kidney function (9, 10) and cardiovascular damage (11), we sought to evaluate how MDR NDM-1 K. pneumoniae (ATCC BAA 2146)-induced pneumonia and/or rCST treatment affected endogenous CST9 and CSTC serum levels. Therefore, we used the archived serum samples of BALB/c mice (Jackson Laboratories) that were infected and/or treated with rCST9/rCSTC (*n* = 6 mice/group) from our previously published studies to quantify endogenous serum CST9 and CSTC via enzyme-linked immunosorbent assay (ThermoFisher and Invitrogen, respectively) (1). All animals were housed in an Association of Assessment and Accreditation of Laboratory Animal Care-approved facility, and procedures were approved by the University of Texas Medical Branch Institutional Animal Care and Use Committee. Our findings revealed that endogenous CST9 and CSTC in the serum samples were significantly higher at 5 days p.i. in untreated infected mice than in mice infected/treated with i.n. and/or i.p. rCST9/rCSTC (Fig. 1A and B; *P* < 0.05). Conversely, i.n. rCST9/rCSTC treatment at 1 h p.i. decreased endogenous serum CST9 and CSTC levels by 72 h, with a significant decrease in CST9 at this time point (*P* = 0.0033). The same mice then received a second dose of rCST9/rCSTC i.p. at 3 days p.i. that resulted in a significant decrease in endogenous CST9 (*P* = 0.0090) and CSTC (*P* = 0.0140) levels at 5 days p.i. compared with untreated infected mice (Fig. 1A and B). Likewise, mice administered a single i.p. dose of rCST9/rCSTC at 3 days p.i. had significantly decreased endogenous CST9 and CSTC levels in the serum at 5 days p.i. (Fig. 1A and B; *P* = 0.0090 and 0.0140, respectively). Note that for Fig. 1A and B, rCST concentrations were not likely detectable in the total measurement of CST9 and CSTC quantified in the serum because of the extremely small dosages, short half-life of cystatins, and timing of the treatment p.i. CSTC is the most studied of the cystatins; however, to our knowledge, there are no published reports regarding endogenous CST9 serum levels. It is known that nucleated cells constitutively produce CSTC, resulting in a stable level of the protein in the blood (9, 10). CSTC is filtered through glomerular filtration, reabsorbed, and metabolized by the proximal tubules (9, 10). If this process is disrupted, CSTC levels are increased in the blood, which can be linked to renal damage (9, 10). Therefore, the rCST9/rCSTC modulation of endogenous CST9, CSTC, and serum amyloid A (SAA) levels in the serum may serve as biomarkers of kidney and liver functions. Our results are the first to show a significant correlation between exogenous rCST9/rCSTC treatment and modulation of endogenous serum CST9 and CSTC levels (Fig. 1), which likely contributed to improved survival outcomes in a mouse model of MDR pneumonia (1).

**FIG 2:**
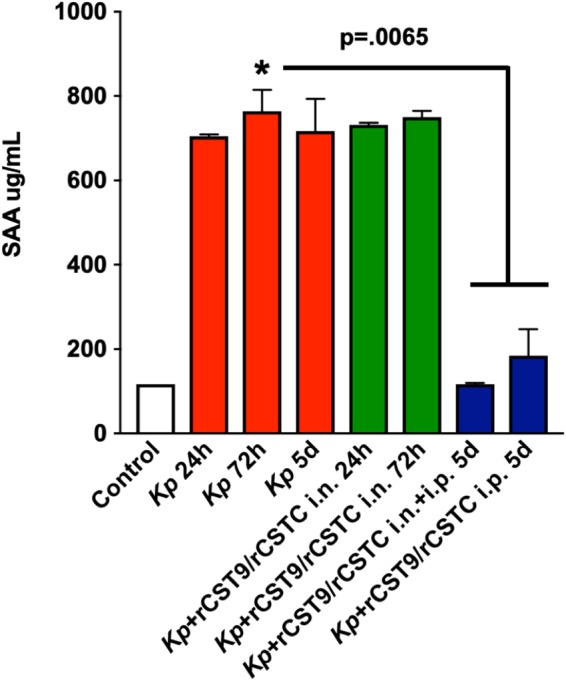
rCST treatment modulated SAA. Both optimal rCST9/rCSTC treatment regimens significantly modulated serum levels of SAA at 5 days after MDR NDM-1 K. pneumoniae infection in treated mice compared with untreated infected mice (*P* = 0.0065). White bars, untreated/uninfected controls; red bars, untreated infected mice; green bars, infected mice receiving rCST9/rCSTC i.n. treatment (50/50 pg) 1 h p.i.; blue bars, infected mice receiving rCST9/rCSTC i.n. treatment (50/50 pg) 1 h p.i. and/or rCST9/rCSTC i.p. (500/500 pg) 3 days p.i. Data are presented as means ± SEM. *, *P* < 0.05.

To begin to correlate rCST treatment with restrained systemic inflammation, we analyzed the same archived mouse serum samples to quantify SAA. SAA is an acute-phase serum protein secreted primarily from the liver that is a biomarker for persistent inflammation (12) and renal damage and is implicated in the induction of enzymes that degrade the extracellular matrix (13). Our results showed that rCST9/rCSTC given i.n. and/or i.p. significantly decreased serum SAA at 5 days p.i., which was equivalent to results in uninfected/untreated control mice, compared with untreated infected mice and infected/rCST-treated mice at 24 and 72 h p.i. (Fig. 2; *P* = 0.0065). The combined data showed that rCST9/rCSTC treatment modulated the levels of endogenous CSTC, CST9, and SAA, which are serum biomarkers that are associated with damaging inflammation (Fig. 1 and 2). The culmination of these findings showed that treatment with rCSTs provided long-term protection that far exceeded their approximate 2-h half-life (9). We are currently in the process of determining the biological mechanisms responsible for the rCST9/rCSTC modulation of endogenous CST9, CSTC, and SAA levels.

**FIG 3:**
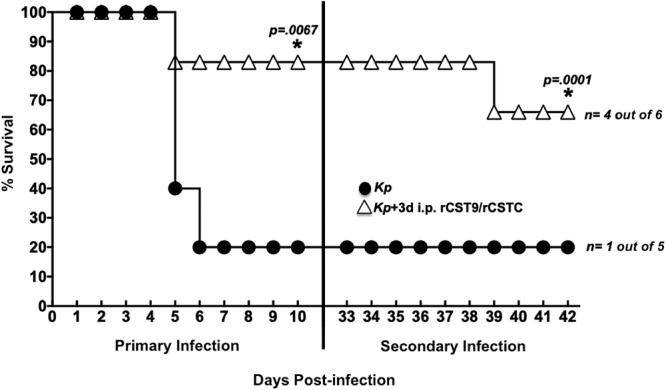
rCST9/rCSTC treatment afforded long-term protection against a second MDR NDM-1 K. pneumoniae challenge in a murine model of pneumonia. BALB/c mice treated with rCST9/rCSTC had significantly increased survival (5 survivors out of 6 mice) compared with untreated mice (1 survivor out of 5 mice; *P* = 0.0067) after primary i.n. LD90 MDR NDM-1 K. pneumoniae challenge. LD90 MDR NDM-1 K. pneumoniae-rechallenged survivors at 33 days p.i. (vertical line) that were initially treated with rCST9/rCTC had only one death by 10 days postrechallenge, versus untreated infected rechallenged mice (*P* = 0.0001). Log-rank analyses with Welch’s corrections via Prism software (GraphPad v7.0c, San Diego, CA) were used to compare survival rates. *, *P* < 0.05.

To determine whether rCST treatment afforded protection against a secondary MDR NDM-1 K. pneumoniae challenge, BALB/c mice that survived an inoculation of LD90 i.n. MDR NDM-1 K. pneumoniae followed by treatment with rCST9/rCSTC (500/500 pg) i.p. 3 days p.i. (1) were rechallenged with NDM-1 K. pneumoniae (LD90) at 33 days p.i. with no additional rCST treatment. The second challenge resulted in four survivors out of six mice initially given rCST9/rCSTC compared with untreated infected mice (one survivor out of five mice; *P* = 0.0001) (Fig. 3).

**FIG 4:**
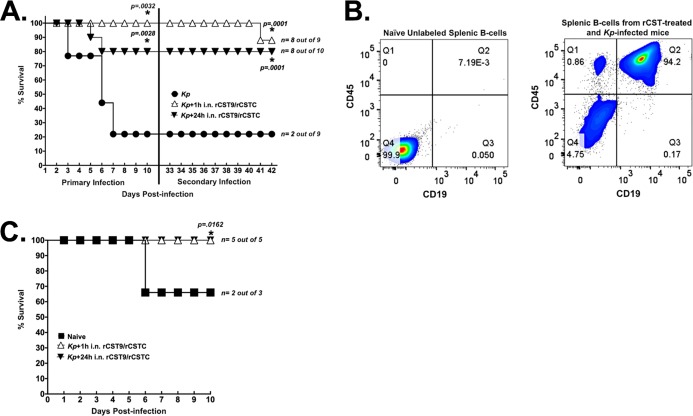
Prophylactic rCST9/rCSTC treatment provided protection against pneumonia, and B cells from prophylactically rCST-treated mice protected naive mice against pneumonia. (A) BALB/c mice coadministered rCST9/rCSTC (50/50 pg) via a single i.n. dose at 1 or 24 h before MDR NDM-1 K. pneumoniae challenge (LD90) showed significantly increased survival (1 h pre-CST, 9 survivors of 9 mice; *P* = 0.0032; 24 h pre-CST, 8 survivors out of 10 mice; *P* = 0.0028) compared with untreated mice (2 survivors out of 9 mice). LD90 MDR NDM-1 K. pneumoniae-rechallenged survivors at 33 days p.i. (vertical line) showed that the 24-h-pretreatment/infected group had 100% survival, and only 1 mouse died in the 1-h-pretreatment/infected group, versus untreated infected mice (*P* = 0.0001). (B) Splenic B cells from rechallenged survivors were labeled with CD19 antibody for B-cell specificity and CD45R/B220 antibody to identify the B-cell specificity via flow cytometry. (C) Treatment-naive BALB/c mice (*n* = 5 mice/group) given B cells from rechallenged prophylactic 24- or 1-h survivors exhibited complete protection against MDR NDM-1 K. pneumoniae pneumonia, demonstrating 100% survival (*P* = 0.0162), versus 2 survivors in the group receiving B cells from naive mice. Log-rank analyses with Welch’s corrections via Prism software (GraphPad, San Diego, CA) were used to compare survival rates. *, *P* < 0.05.

Furthermore, a one-time i.n. prophylactic dose of rCST9/rCSTC (50/50 pg) at 24 h (*n* = 10 mice) or 1 h (*n* = 9 mice) p.i. significantly improved survival outcomes against a primary pneumonia infection and a secondary rechallenge with a dose of LD90 MDR NDM-1 K. pneumoniae in treated mice compared with untreated infected mice (*P* = 0.0001) (Fig. 4A). We expanded this study with an adoptive transfer of splenic B cells from the 24- or 1-h prophylactically rCST-treated/infected mice to treatment-naive mice (1.2 × 10^6^ B cells i.v./mouse). Collection of isolated B cells from survivors >8 weeks posttreatment and infection ensured that they were memory B cells (14–16) (Fig. 4B). One-day after adoptive B-cell transfer, mice were inoculated with LD90 NDM-1 K. pneumoniae i.n. Mice receiving B cells from the 24- or 1-h pre-rCST treatment showed 100% protection against MDR NDM-1 K. pneumoniae, whereas 33% of mice that received naive B cells succumbed to infection (*P* = 0.0162) (Fig. 4C). Results from the rCST treatment p.i. and prophylactic studies lend further evidence that coadministration of rCST9/rCSTC is not transient but has synergistic, lasting effects on the host’s immune responses beyond the rCST 2-h half-life (1). These data further demonstrate that rCST9/rCSTC efficacy to combat a primary infection appears not to impair humoral immune responses against a secondary infection (1, 9). As we observed with our rCST treatments, immune responses are beneficial and balanced, allowing for the robust development of innate and adaptive immunity to fight against a primary and secondary pneumonia infection. Alternatively, in some cases, rapid clearance of bacteria and associated antigens after antibiotic treatment can have consequential effects that impair CD4 T-cell memory, leading to incomplete humoral immune development, which results in a lack of protection against subsequent infections (17, 18).

There are no published findings to help elucidate why exogenously administered rCSTs would promote prophylactic and/or long-term protection against pneumonia. However, it is reasonable to hypothesize that the multifaceted functions of rCST9/rCSTC treatment promote cell survival and controlled beneficial immune responses against pneumonia (1). This, in turn, would increase the overall health of the host, allowing for the allocation of resources to bolster the humoral immune response, subsequently improving survival outcomes. We do not want to overinterpret or overstate the effects of rCST9/rCSTC treatment but offer rCST9/rCSTC as a promising alternative treatment for pneumonia to be used alone or combined with less-toxic doses of appropriate antibiotics (1).

## References

[1] Holloway A, Yu J, Arulanandam B, Hoskinson S, Eaves-Pyles T 2018 Cystatin 9 and C: a novel immunotherapy that protects against multidrug-resistant New Delhi metallo-beta-lactamase-1-producing *Klebsiella pneumoniae*. Antimicrob Agents Chemother 62:e01900-17. doi:10.1128/AAC.01900-17. 29229643 PMC5826106

[2] Zavasnik-Bergant T 2008 Cystatin protease inhibitors and immune functions. Front Biosci 13:4625–4637. 18508534 10.2741/3028

[3] Ochieng J, Chaudhuri G 2010 Cystatin superfamily. J Health Care Poor Underserved 21:51–70. doi:10.1353/hpu.0.0257. 20173285 PMC2888135

[4] Bobek LA, Levine MJ 1992 Cystatins—inhibitors of cysteine proteinases. Crit Rev Oral Biol Med 3:307–332. doi:10.1177/10454411920030040101. 1391414

[5] Kopitar-Jerala N 2006 The role of cystatins in cells of the immune system. FEBS Lett 580:6295–6301. doi:10.1016/j.febslet.2006.10.055. 17098233

[6] Poteryaeva ON, Falameyeva OV, Korolenko TA, Kaledin VI, Djanayeva SJ, Nowicky JW, Sandula J 2000 Cysteine proteinase inhibitor level in tumor and normal tissues in control and cured mice. Drugs Exp Clin Res 26:301–306. 11345042

[7] Vray B, Hartmann S, Hoebeke J 2002 Immunomodulatory properties of cystatins. Cell Mol Life Sci 59:1503–1512. doi:10.1007/s00018-002-8525-4. 12440772 PMC11337455

[8] Eaves-Pyles T, Patel J, Arigi E, Cong Y, Cao A, Garg N, Dhiman M, Pyles RB, Arulanandam B, Miller AL, Popov VL, Soong L, Carlsen ED, Coletta C, Szabo C, Almeida IC 2013 Immunomodulatory and antibacterial effects of cystatin 9 against *Francisella tularensis*. Mol Med 19:263–275. 23922243 10.2119/molmed.2013.00081 PMC3769527

[9] Jeon YK, Kim MR, Huh JE, Mok JY, Song SH, Kim SS, Kim BH, Lee SH, Kim KY, Kim IJ 2011 Cystatin C as an early biomarker of nephropathy in patients with type 2 diabetes. J Korean Med Sci 26:258–263. doi:10.3346/jkms.2011.26.2.258. 21286018 PMC3031011

[10] Hojs R, Bevc S, Ekart R, Gorenjak M, Puklavec L 2006 Serum cystatin C as an endogenous marker of renal function in patients with mild to moderate impairment of kidney function. Nephrol Dial Transplant 21:1855–1862. doi:10.1093/ndt/gfl073. 16524933

[11] Angelidis C, Deftereos S, Giannopoulos G, Anatoliotakis N, Bouras G, Hatzis G, Panagopoulou V, Pyrgakis V, Cleman MW 2013 Cystatin C: an emerging biomarker in cardiovascular disease. Curr Top Med Chem 13:164–179. doi:10.2174/1568026611313020006. 23470076

[12] Ni XC, Yi Y, Fu YP, He HW, Cai XY, Wang JX, Zhou J, Fan J, Qiu SJ 2014 Serum amyloid A is a novel prognostic biomarker in hepatocellular carcinoma. Asian Pac J Cancer Prev 15:10713–10718. 25605163 10.7314/apjcp.2014.15.24.10713

[13] Preciado-Patt L, Hershkoviz R, Fridkin M, Lider O 1996 Serum amyloid A binds specific extracellular matrix glycoproteins and induces the adhesion of resting CD4+ T cells. J Immunol 156:1189–1195. 8557997

[14] Jones DD, Wilmore JR, Allman D 2015 Cellular dynamics of memory B cell populations: IgM+ and IgG+ memory B cells persist indefinitely as quiescent cells. J Immunol 195:4753–4759. doi:10.4049/jimmunol.1501365. 26438523 PMC4637268

[15] Hoffman W, Lakkis FG, Chalasani G 2016 B cells, antibodies, and more. Clin J Am Soc Nephrol 11:137–154. doi:10.2215/CJN.09430915. 26700440 PMC4702236

[16] McHeyzer-Williams LJ, McHeyzer-Williams MG 2005 Antigen-specific memory B cell development. Annu Rev Immunol 23:487–513. doi:10.1146/annurev.immunol.23.021704.115732. 15771579

[17] Benoun JM, Labuda JC, McSorley SJ 2016 Collateral damage: detrimental effect of antibiotics on the development of protective immune memory. mBio 7:e01520-16. doi:10.1128/mBio.01520-16. 27999159 PMC5181774

[18] Benoun JM, Labuda JC, Fogassy ZN, Pham O, Pham QM, Puddington L, McSorley SJ 2017 Antibiotic treatment causes a reduction in antigen-specific T cell memory and increased susceptibility to secondary infection. J Immunol 198(Suppl 1):216.8.

